# Experience and memory of time and emotions two years after the start of the COVID-19 pandemic

**DOI:** 10.1371/journal.pone.0290697

**Published:** 2023-09-20

**Authors:** Sylvie Droit-Volet, Natalia Martinelli, Guillaume Dezecache, Clément Belletier, Sandrine Gil, Johann Chevalère, Pascal Huguet

**Affiliations:** 1 CNRS, LAPSCO, Université Clermont Auvergne, Clermont-Ferrand, France; 2 CNRS, UMR 7295, Centre de Recherches sur la Cognition et l’Apprentissage, Université de Poitiers, Poitiers, France; University of Padova, ITALY

## Abstract

In this French longitudinal study, we assessed judgment of the passage of time in current life and the predictors of this judgment 2 years after the onset of the COVID-19 pandemic, i.e., at a time when there was no lockdown and no protective measures. We then compared these measures with the same participants’ passage-of-time judgments assessed during each of the past three French lockdowns. We also assessed their memory representations of the passage of time in the past, i.e., for the various lockdowns. The results showed the persistence of the feeling of time slowing down outside of lockdown. However, this was no longer linked to external factors (lack of activity, disruption of everyday routines) as found in the previous studies conducted during the lockdowns, but to an individual internal factor, namely a high level of depression in the general population. Moreover, the results revealed that the experience of the passage of time for the past lockdowns was compressed in memory, being judged to be faster than it actually was. This time compression tended to be greater in depressed people. It was also associated with a positive bias for all the other examined factors (e.g., sleep quality, life routine, boredom, happiness). We assumed that this time compression would be related to processes involved in the recall of unfolding events, with certain moments being omitted or forgotten during recall, as well as to the process of reconstruction in autobiographical memory. Our study therefore shows the long-lasting effect of lockdowns on mental health of the general population, which was expressed by the persistent feeling of a slowing down of time. It is therefore necessary to take care of this psychologically fragile population and to avoid further lockdowns in response to a new health crisis, that they cannot cope with.

## Introduction

In 2020, governments in many countries recognized the COVID-19 pandemic as a major health crisis which required their respective populations to be confined at home for periods of several weeks and/or months. People were restricted to their homes, where they were bored, socially isolated from their loved ones and deprived of freedom. This confinement at home resulted in a modification of their representation of the passage of time (PoT). They experienced a slowing down of time during the lockdown compared to before it [1–4]. Studies conducted around the world (Brazil, Canada, Italy, Iraq, France, Germany, United Kingdom, Uruguay) [5–10, for a database including different countries, see 11] confirmed this cross-cultural phenomenological experience. However, in Argentina, more participants reported an acceleration rather than a slowing of PoT during the lockdown [12]. This observation rules out neither a role of cultural and economic factors nor that of differences in confinement measures and their monitoring between countries.

According to the contextual self-duration theory of PoT judgment in the present, variations of this temporal judgment are based on perceived changes in each individual’s specific internal context (e.g., negative affect) and the environment-specific external context (e.g., lack of activity, social isolation) [13–15]. It might therefore seem reasonable to state that the judgment that time passed more slowly during the lockdown period was mainly due to changes in the external context, i.e., changes resulting from lockdown-related events and their effects on individuals’ subjective experiencing. Studies that have sought to identify the predictors of PoT judgments have shown that fear for one’s own health and that of loved ones or stress regarding the pandemic did not explain the feeling of a slowing-down of the PoT [1–4, 7]. Instead, this temporal feeling was due to the life conditions linked to the lockdown, which were considered unsatisfactory, in particular because people were socially isolated and their daily routines disrupted. Indeed, the impression of a slowing down of time increased, the more socially isolated and lonely people were [6, 11]. Loss of daily routine (work, leisure activities), disruption of sleep, and high levels of negative emotions (boredom and sadness), leading to decreased life satisfaction, were also identified as different significant predictors of changes in the sense of time for the first lockdown [1–4, 7, 10].

Thanks to their coping abilities, most people were nevertheless gradually able to adapt to these new living conditions [10, 16]. After a few weeks of lockdown, they slept better, developed new daily routines and felt happier. However, distortions in time judgment remained despite these adaptations. Studies examining the changes in participants’ PoT judgments during the lockdown period showed that time was consistently considered to be slow-moving during the weeks of lockdown, with no significant changes in mean temporal scores being observed over a period of 6 weeks in the French study [16], and with only a slight acceleration over a longer period of 14 weeks and 15–26 weeks in a Brazilian [6] and a German study [10], respectively. However, this persistent feeling of a slowing-down of time can be explained by the fact that individuals were still confined.

If external factors related to the life conditions of lockdown fully accounted for the feeling of a slowing-down of time during the lockdown, the perceived flow of time should logically have quickly returned to its usual pace after the lockdown. However, waves of the COVID-19 pandemic have followed one another and there have been recurrent periods of lockdown. In France, three lockdown periods occurred during a full year, from 2020 to 2021. A longitudinal follow-up of French participants showed that they suffered significantly less as each lockdown succeeded the last [16] and the lockdown rules became less stringent. However, even though the experience of lockdown became less negative with habituation and with the accompanying relaxation of the rules, participants still considered time to pass more slowly than before the first lockdown [16]. Indeed, no significant difference in mean temporal scores was observed between the three lockdown periods covering one whole year in this French study. Similarly, a survey conducted in the United Kingdom 8 months and 12 months after the first lockdown found that the experience of the time that had passed since the first lockdown was “highly skewed towards a slowing”, despite the relaxation of restrictive confinement rules [17, 18]. A recent study in Iraq confirmed the persistence of a sense of slow time 11 months after the first lockdown [5]. However, a German study has highlighted inter-individual differences, with many participants reporting that the last 14 months since the beginning of the pandemic seemed to be longer than normal, whereas others reported that they appeared shorter. In summary, the sense that time passes more slowly in the present than before the pandemic seems to persist long after the first lockdown for many participants, regardless of changes in living context, i.e., improved living conditions.

A large number of factors have been identified as significant predictors of the slowing of the passage of time for the first lockdown (e.g., boredom, happiness, sleep, life rhythm, life satisfaction), with depression playing a relatively minor role [2–4, 10]. However, French and British surveys indicated that the individual level of depression had become a major predictor of PoT judgment one year after the first lockdown, with boredom and social dissatisfaction still having a significant effect [16–18]. It can be assumed that the significant effect of the latter two factors (boredom, social dissatisfaction) on temporal judgments was directly linked to life circumstances, since the lockdowns (2nd and 3rd) were still in place at the time of these surveys. However, if the feeling of time passing slowly persists in the long term when no lockdown is in place, then the general affective state of depression should remain the only reliable factor explaining this temporal feeling.

The persistent feeling of a slowed-down time out of lockdown would thus indicate the emergence of chronic depressive symptoms in the population, which exist independently of the improvement of living conditions or of the reduction of boredom due to the wider range of activities available, for example. Permanent impairment of time perception could be an indication of low levels of well-being characteristic of the development of a chronic psychiatric disorder [19]. Many international studies have highlighted the long-lasting effect of lockdowns on mental health, with an increase in the number of depressed people in the general population [20, 21]. According to clinical studies, depressed people and those who have experienced trauma do indeed have an altered perception of time and experience the feeling that time has stopped or passed very slowly [22–24].

The PoT judgment in the present thus depends on the perception of the current external or internal context, e.g., the depressive emotional state outside of lockdown. However, like all judgments, the PoT judgment results from a system of information processing composed of different cognitive levels, including memory and decision processes. It therefore also depends on memory processes, i.e., the recollection of the speed of the passage of time experienced in the past. If the passage of time associated with past events is judged to have been very fast, then, in comparison, the passage of time associated with present events might be judged to be relatively slower. Thus, processes which compare the past PoT in memory with the present PoT could impact the PoT judgment that participants report when surveyed.

Apart from a few laboratory studies, little research has been devoted to the long-term memory of time associated with real-life events. Recently, Jeunehomme et al. [25–27] demonstrated a temporal compression of events in episodic memory, showing, for example, that the remembered duration of past events is typically shorter than their actual duration. Memories of the speed of the passage of time for specific past events might therefore become distorted, the older these memories are, with time during the previous lockdown periods being judged as having passed faster than it actually did. As reported above and discussed later, only a few COVID studies have examined the retrospective judgment of PoT, compared to the usual PoT judgment, for a long interval spanning several months since the beginning of the pandemic or the first lockdown (8, 11, 12, 14 months) [5, 7, 17, 18] and, to our knowledge, none has studied the recall of the current PoT experienced during the past periods of lockdown. To investigate the long-term memory of the PoT, we therefore not only assessed the participants’ judgment of the PoT in the present (outside lockdown), but also its recollection in memory for each of the three successive lockdown periods in France. The value of our longitudinal study, with the same participants being surveyed during each lockdown, is that it gives us the opportunity to directly compare the memory of PoT for each lockdown period with the PoT actually experienced during each lockdown.

Therefore, the first aim of the present study was to examine whether the feeling of a slowing down of time observed during the lockdown periods persists outside of the lockdown, and, if it persists, what is the factor that best predicts this temporal feeling when participants resume their normal lives, i.e., lives similar to those they led before the pandemic. Our survey was thus carried out in France outside of lockdown and in the absence of any protective measures. More specifically, it was conducted in June 2022, i.e., two years after the first lockdown and one year after the third and last lockdown. Our hypothesis (hypothesis 1) was that the persistence of a slowdown in the perceived passage of time outside the specific context of life during lockdown is linked to the level of depression in the general population. The major predictor of the feeling of slowed-down time outside of lockdown would therefore be the individual level of depression and no longer the factors identified during the first period of lockdown (quality of sleep, life rhythm, boredom, happiness). Therefore, in our study, we assessed the feeling of the passage of time in the present outside lockdown and compared it with that recorded during the different lockdown periods. We also assessed the potential predictors of this feeling, namely level of depression, quality of sleep, life rhythm, boredom, happiness (i.e., the significant predictors found in previous French studies).

The second aim of our study was to examine the recall of the PoT experience during past periods of lockdown. There is indeed no study on the memory of passage of time. However, the examination of memories about the feeling of the passage of time in the past is important to appreciate, in particular, whether there are reconstructions in autobiographical memory revealing defense strategies in the face of “traumatic” confinement events. It was expected (hypothesis 2) that a distortion in the memory representation of the passage of time (time compression) would occur for the past periods of lockdown and that this would increase, the older the memories are, in turn magnifying the feeling that time is passing slowly in the present. In our longitudinal study, we therefore assessed participants’ judgment of the PoT in the present outside of lockdown (two years after the first lockdown) and its recall in memory for each of the three prior French lockdowns. We then compared these recalled values with the participants’ actual judgments that we had recorded during each of three lockdown periods.

## Material and methods

### Participants

The final sample consisted of 469 participants who agreed to respond to our survey in June 2022, outside lockdown (241 men, 228 women; *M*_Age_ = 51.39, *SD* = 14.07, *M*_Education years_ = 12.13, *SD* = 3.22). These participants also responded to our survey 3 times before, at each of the 3 lockdowns in France (April 2020, November 2020, April 2021). The final sample represented 43% of the participants of the initial sample (April 2020, *N* = 1082). There were not significantly differences (e.g., anxiety scores, depression scores, etc.) between the initial and the final sample of participants except that the latter were older (50.10 vs. 44.14, *t*(1080) = 6.62, *p* < .001, Cohen’s *d* = 0.41). Participants were all major and recruited by a survey company (EasyPanel), which gave them a voucher to reward them for their participation. Participants read an informed consent form which explained that they could decide not to participate and stop the survey at any time. Then, a question with a “yes/no” answer was presented: “I have read and understood the above information and I agree to participate in this study”. A “no” answer did indeed screen out the participants. Answering this question was considered as consent by the research ethics committee of the Clermont Auvergne. The need for written or oral consent was therefore waived by the ethics committee which approved this longitudinal study (MRA-19/20-18273), which followed the principles of the Declaration of Helsinki. Anonymous data were hosted on the local server of Clermont Auvergne University (France).

### Procedure

Participants completed the online survey 4 times: at *T*1 (24–28 April 2020) during the first lockdown imposed in France, *T*2 (12–18 November 2020) during the second lockdown, *T*3 (6–12 April 2021) during the third lockdown, and *T*4 (30 May—6 June 2022) out of lockdown. The last survey was carried out during a period with no wave of COVID-19 and its variants, and in the absence of health measures (e.g., mandatory wearing of masks). The last survey was therefore carried out approximately 2 years after the first lockdown and 1 year after the third and last lockdown. Among a range of other questions, it contained the same questions with a 7-point response scale as those asked in the 3 previous surveys: i.e., questions on the experience of the PoT (“Now, the speed of the passage of time seems to me to go from 1 ‘*very slow’* to 7 ‘*very fast’*), and its significant predictors: quality of sleep, life rhythm, boredom, happiness (“Now, I sleep well/the rhythm of my life was regular (getting up, eating, going to bed)/I am bored/I feel happy) from 1 *‘not at all’* to 7 *‘a lot/completely’* [3, 16]. The survey also contained the clinical scales used in previous surveys, namely the depression scale (Beck Depression Inventory, BDI) [28], and the anxiety scale (Short State Trait Anxiety Inventory, S-STAI) [29], the scores on which were also found to be significantly correlated with the PoT judgment (correlation metrics and associated *p* values). The reliability of these two multiple-item scales was always good (α_*T*4_ = 0.908, α_*T*4_ = 0.905, respectively).

A series of other questions were added to our survey given at *T4* on the memory of the judgment of the passage of time during each lockdown, i.e., at *T1*, *T2* and *T3*, and before the first lockdown: “Before the first lockdown/During the first lockdown (March-April 2020)/During the second lockdown (October-December 2020)/ During the third lockdown (April 2021), the speed of the passage of time seemed to me to go from 1 ‘*very slow’* to 7 ‘*very fast’*”. Participants also had to recall their experiences (sleep quality, life rhythm) and emotions (Boredom, Happiness, Sadness, Fear, Anger, Low-arousal, High-arousal, Anxiety) before the first lockdown and during each lockdown.

First, a series of statistical analyses (ANOVA, *t*-tests, and analyses based on linear regressions) were carried out to test changes in the experience of the passage of time over two years, from the first (April 2020), second (November 2020) and third lockdown (April 2021) to now (June 2022), but also those in the factors initially identified as significant predictors of the PoT judgment (quality of sleep, life rhythm, boredom, happiness, anxiety, and depression) [3, 16] (see S1 Dataset). We then tested their relationships to identify the major predictor of the current PoT judgment. Second, using similar statistical analyses, we tested the memory recall of the PoT judgment and judgment of other factors for the different lockdown periods in the past, which we compared to the actual judgment previously recorded at each lockdown.

## Results

### Experience of the passage of time and its evolution over time

**Fig 1:**
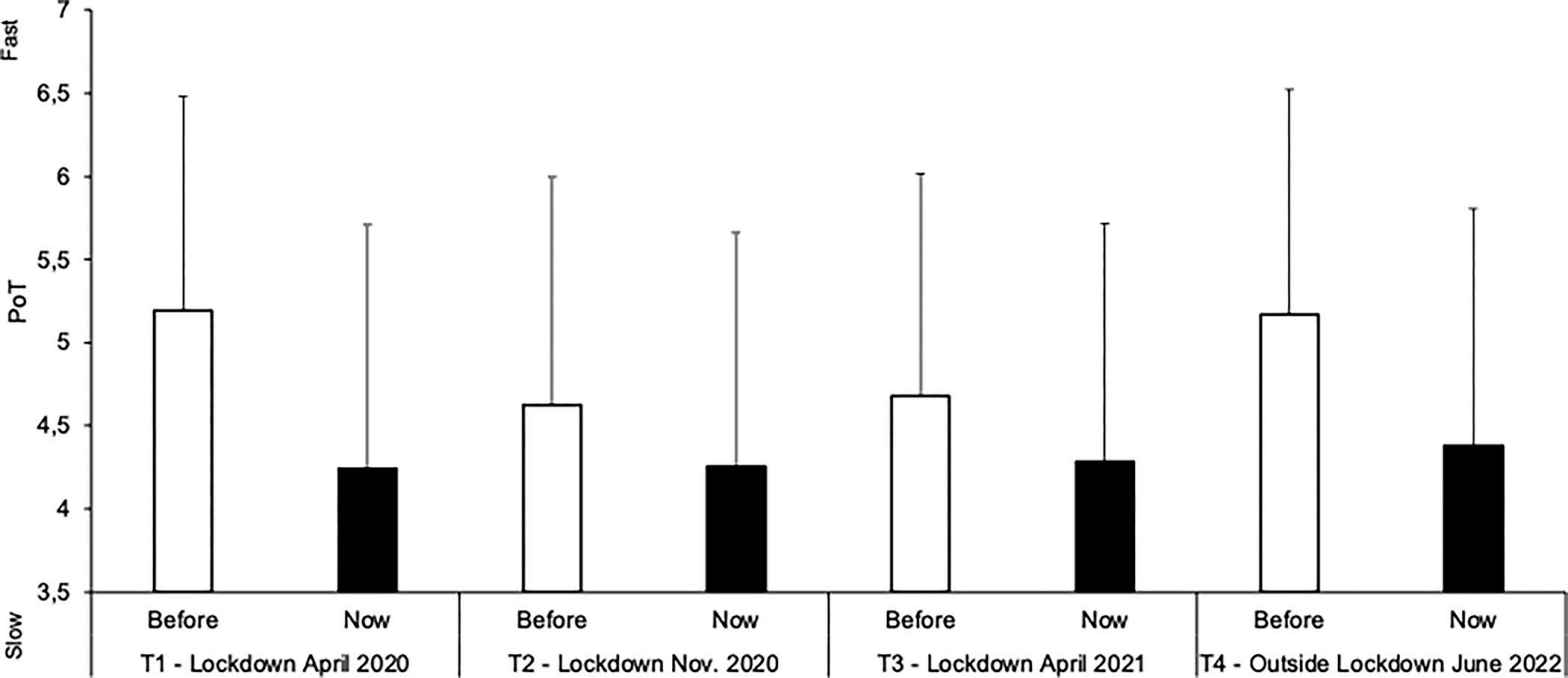
Passage of time. Mean (SD) Passage-of-Time (PoT) judgments (from 1 “very slow” to 7 “very fast”) for the present-PoT judgment (Now) and the recall of PoT before the first lockdown (Before) recorded at each survey time (*T1*, *T2*, *T3* and *T4*).

Fig 1 shows the present PoT (Now) recorded at *T*4, in June 2022 (1 year after the last lockdown), and at *T*1, *T*2, and *T*3 from April 2020 to April 2021 (i.e., during each lockdown), compared to the recall of the PoT before the first lockdown (Memory-of-before), which was evaluated during each survey. An ANOVA was performed on the PoT judgment with two within-participants factors: 1) Now/Memory-of-before; 2) Survey time (*T*1*/T*2*/T*3*/T*4). This ANOVA showed a highly significant main effect of the Now/Memory factor, *F*(1, 468) = 185.76, *p* < .001, η^2^_p_ = 0.28, indicating that the present PoT has always been judged slower than the recalled PoT for the period before the pandemic, i.e., regardless of the time elapsed since the pre-pandemic period (*M*_Now_ = 4.29, *SD* = 1.43; *M*_Memory-of-before_ = 4.92, *SD* = 1.34). Even one year after the last lockdown (*T*4), the PoT continued to be judged to be slower than the recalled pre-pandemic PoT (*M*_*T*4-Now_ = 4.38, *SD* = 1.42; *M*_*T*4*-Memory-of-before*_ = 5.17, *SD* = 1.35, *t*(468) = 11.13, *p* < .001, Cohen’s *d* = 0.51). Therefore, participants considered that the speed of the passage of time had not recovered its initial pace two years after the beginning of the pandemic and continued to judge it as slower than before the COVID-19 pandemic.

The ANOVA nevertheless revealed a significant interaction between the Now/Memory-of-before and the survey time factor, *F*(3, 1404) = 18.57, *p* < .001, η^2^_p_ = .04, with an underlying main effect of survey time, *F*(3, 1404) = 16.80, *p* < .001, η^2^_p_ = .04. This interaction indicated that the contrast between the recollection of time lived before the pandemic and the experience of time in the present differed depending on when participants responded to the survey. We thus calculated an index of the difference between the judgment of the passage of time now and before the pandemic. A repeated ANOVA was then performed on this index with the survey time as within-participant factor (*T*2, *T*3, *T*4). We did not include *T*1, when the contrast between the PoT before and during the lockdown was particularly large. This ANOVA indicated that the contrast between the present PoT and the PoT remembered before the pandemic increased, the older the memory of this pre-pandemic PoT was, i.e. from *T*2 to *T*4, *F*(1, 468) = 20.22, *p* < .001, η^2^_p_ = 0.04. The Now/Memory-of-before difference was indeed higher at *T*4 (M = 0.79) than at *T*3 (*M* = 0.40) or *T*2 (*M* = 0.37) (*t*(468) = 4.24, *p* < .001, *d* = 0.20; *t*(468) = 4.50, *p* < .001, *d* = 0.21, respectively). No significant difference was observed between T4 and T1, *t*(468) = -1.67, *p* = .097, *d* = -0.08. The increase with survey time of the difference between judgment of the PoT for the present and that recalled for the pre-pandemic period is mainly explained by a memory age-related distortion in the representation of pre-pandemic PoT, whereas the judgment of PoT for the present did not change from one survey to the next.

For recall of PoT before the pandemic, we did indeed find a quadratic effect of survey time, *F*(1, 468) = 95.95, *p* < .001, η^2^_p_ = 0.17, with a faster time being recalled at *T*1 (immediate recall) and *T*4 (most distant deferred recall). Between *T*2 and *T*4, time was compressed in long-term memory as the interval between the time of the memory and its recall increased. At *T*4, the PoT before the pandemic was therefore judged to have gone faster (*M*_*T*4-Memory-of-before_ = 5.17, *SD* = 1.35), than when the same judgment was made at *T2* (*M*_*T*2-Memory-of-before_ = 4.62, *SD* = 1.373), or *T*3 (*M*_*t*3-Memory-of before_ = 4.68, *SD* = 1.336) (*t*(468) = 7.417, *p* < .001, *d* = 0.342; *t*(468) = 6.42, *p* < .001, *d* = 0.296, respectively). Consequently, at *T*4, the memory of the speed of the passage of time before the pandemic returned to a level similar to that observed at *T*1 (*M*_*T*1-Memory-of-contrast_ = 5.20, *SD*_*t*1_ = 1.288), *t*(468) = -0.364, *p* = 0.72, *d* = -0.017.

By contrast, for the judgment of the PoT in the present recorded at each survey time, there was no significant change in temporal judgment between the different survey times, from *T*1 to *T4* (*M*_*T*1-Now_ = 4.24, *SD* = 1.469; *M*_*T*2-Now_ = 4.26, *SD* = 1.405; *M*_*T*3-Now_ = 4.29, *SD* = 1.428; *M*_*T*4-Now_ = 4.38, *SD* = 1.423; *F*(3, 1404) = 1.41, *p* = 0.24). Therefore, on average, the speed of the passage of time experienced in daily life had not returned to its initial rate two years after the first lockdown.

**Fig 2:**
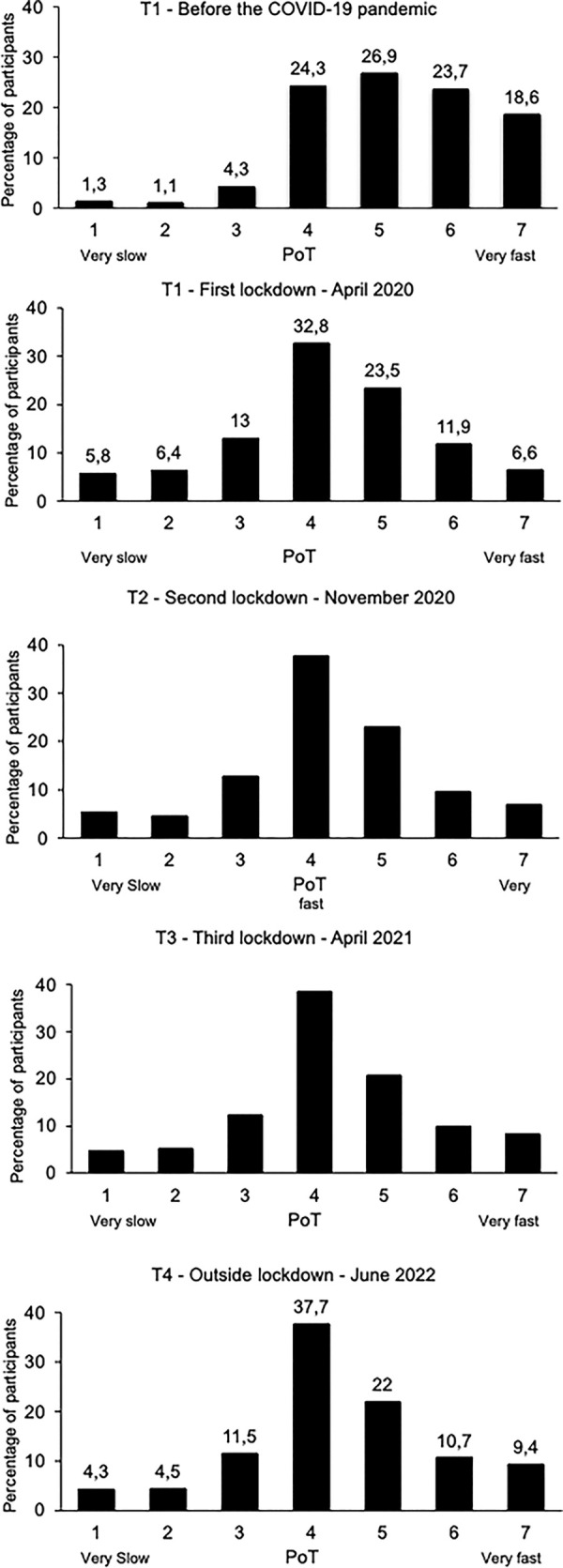
Participants and passage of time. Distribution (in percentage) of participants based on their judgment of the Passage of Time (PoT) on the 7-point scale from 1 “very slow” to 7 “very fast” for each survey time.

Fig 2 shows the distribution of participants according to their PoT judgment. It confirms that before the COVID-19 pandemic (*T*1), time was judged to pass very quickly (> 4 on a 7-point scale) by 69.2% of participants. And 2 years after (*T*4), only 42.1% of participants still considered that time passed quickly (-27%). At *T*4, 20.3% of participants felt that the pace of time was very slow (< 4), compared to only 6.7% before the pandemic.

**Fig 3:**
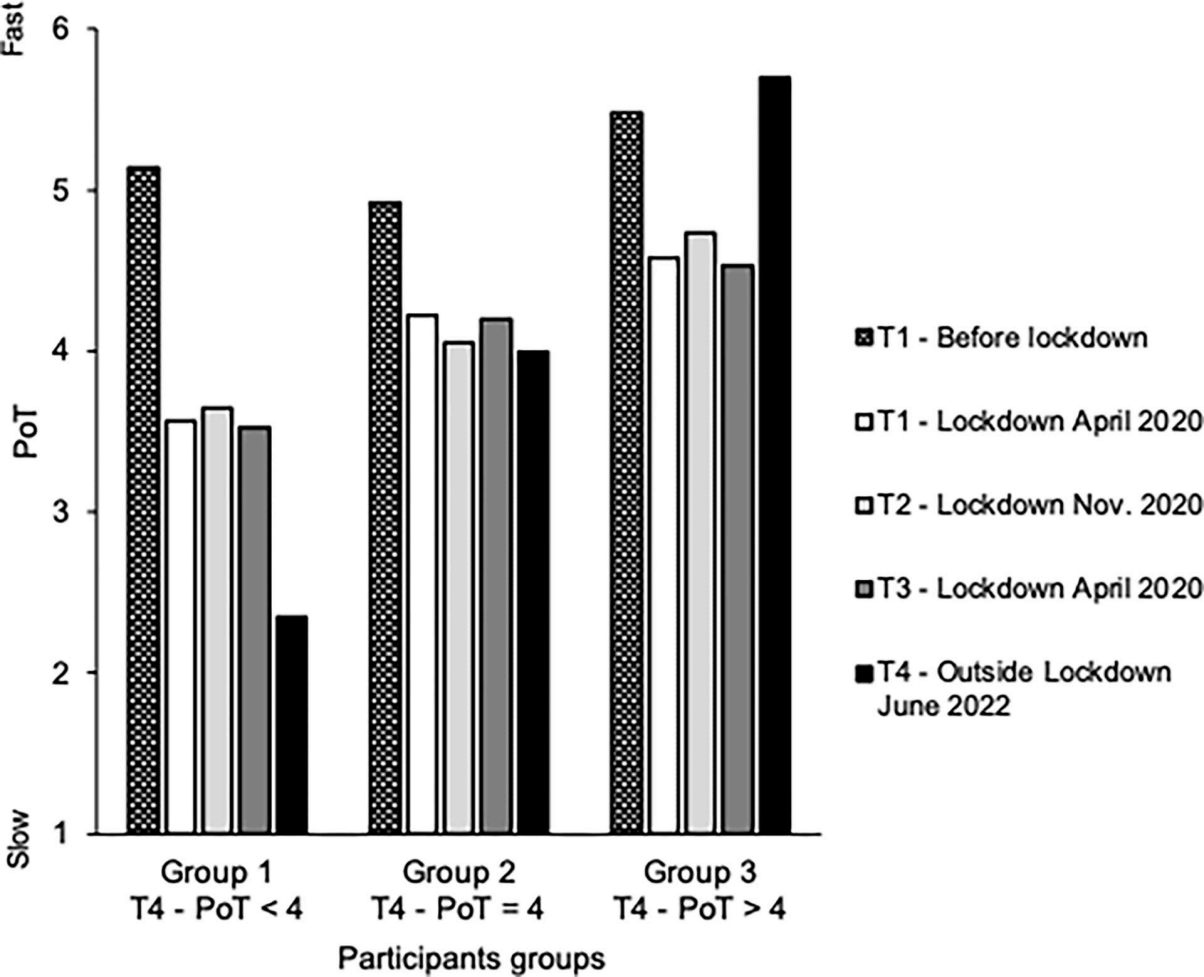
Temporal categories of participants. Judgment of the Passage of Time at the different survey times for the participants who, 2 years after the beginning of the COVID-19 pandemic, i.e., at T4 in June 2022, judged the passage of time as slow (< 4) (group 1), neither fast nor slow (= 4) (group 2) and fast (> 4) (group 3).

Fig 3 shows the PoT judgment collected at each survey time (*T*1, *T*2, *T*3, *T*4) for the participant groups who, at *T*4, reported either a slow PoT (group 1, PoT < 4), a fast PoT (group 3, PoT > 4) or an intermediate PoT (group 2, PoT = 4). It appears that participants who experienced a slow PoT at *T*4 (group 1) had a sense of the passage of time before the pandemic (*T*1- before lockdown) similar to that reported by participants who experienced a fast PoT at *T*4 (group 3) (5.14 vs. 5.48, *t*(289) = 1.99, *p* = 0.5). However, during the first lockdown (*T*1- April 2020), they experienced a stronger sense of time slowing down than the others (3.56 vs. 4.58, *t*(289) = 5.32, *p* < .001, *d* = 0.67). Their feeling of a slowdown in time also increased from each survey to the next and the PoT was reported to be slower at T4 than at *T*1 (2.25 vs. 3.56, *t*(93) = 8.15, *p* < .001, *d* = 0.84). By contrast, the other participants (group 3) experienced a significant acceleration of time between *T*1 and *T*4 (5.70 vs. 4.58, *t*(196) = 9.10, *p* > .001, *d* = 0.65). The participants with an intermediate PoT judgment at *T*4 (group 2) also experienced a slowing-down of time from *T*1 to *T*4 (4.22 vs. 3.99, *t*(177) = 2.47, *p* = .01, *d* = 0.19), although of smaller amplitude than those in the first group. In line with the previous results calculated using mean data, these results indicate that the feeling of the slowing of time persisted in most participants (58%). A gradual return to the pre-pandemic pace of time (acceleration of time) was nevertheless observed in a smaller proportion of participants (42%). Despite this, the speed of PoT had not yet reached its pre-pandemic pace even at *T*4.

### Predictors of passage of time and their evolution over time

**Fig 4:**
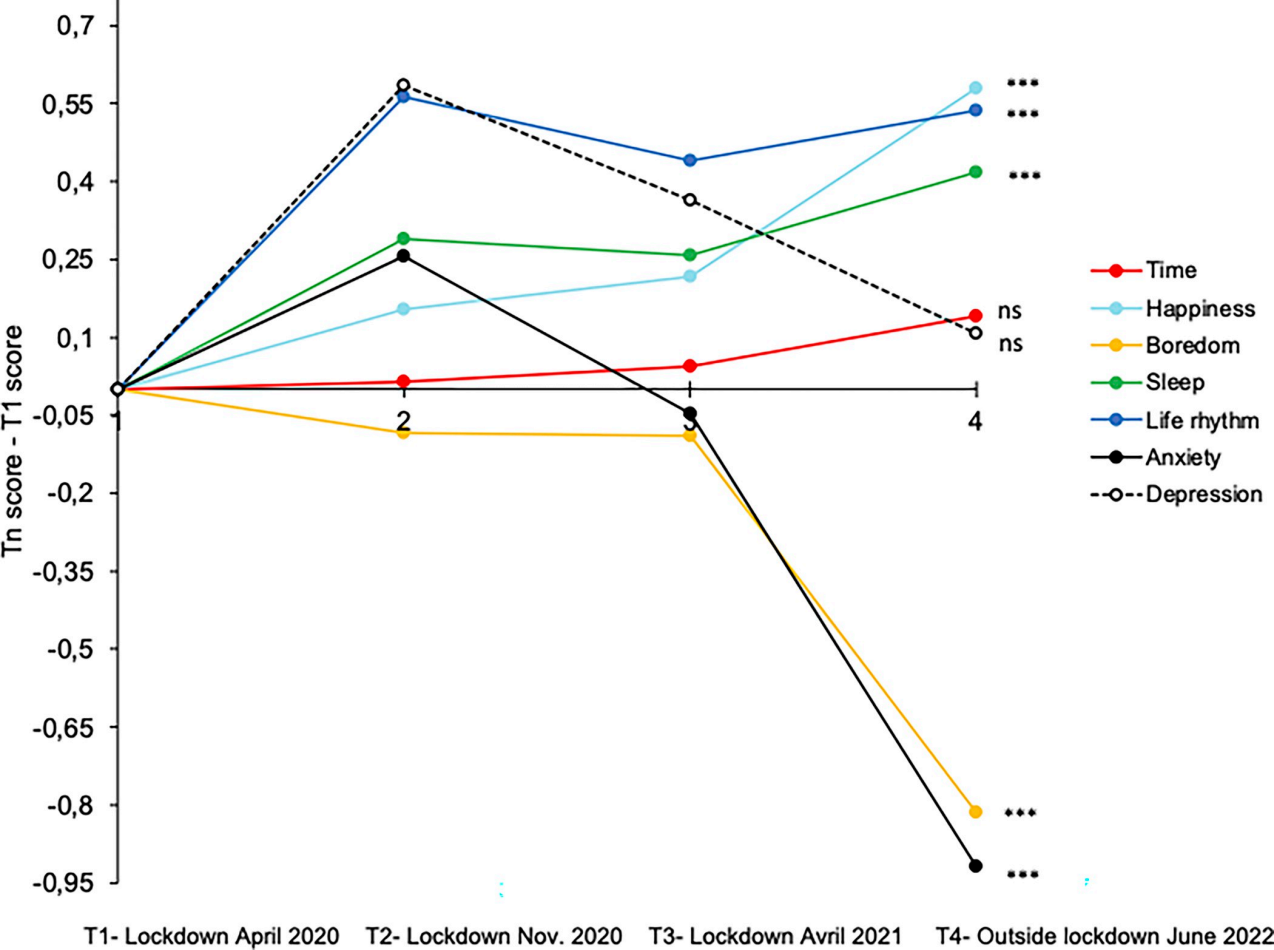
Evolution of judgments. Differences between the score obtained in the different surveys and the score obtained during the first survey (first lockdown) for different factors: time, happiness, boredom, sleep, life rhythm, anxiety, depression.

Fig 4 illustrates the evolution over the different survey times (from *T*1 to *T*4) of different factors that were identified as predictors of passage of time during the first lockdown [3, 16]: quality of sleep, life rhythm, happiness, boredom, anxiety and depression. The evolution of the present PoT judgment is also indicated in Fig 4. To account for the changes in these factors, we calculated a difference index between the score obtained at each survey time (*T1*, *T*2, *T*3 or *T*4) and the initial score at *T*1 (*T*n score—*T*1 score). We then performed an ANOVA on this index for each factor, with the survey time (*T1/T*2*/T*3*/T*4) as within-participants factor. Pairwise comparisons between the scores at *T*3 (during the last lockdown) and *T*4 (one year after) were also carried out. The results revealed a significant improvement for all factors, with the exception of depression scores, as was also the case for the present PoT judgment as reported above.

The ANOVA did indeed show a linear improvement for happiness, *F*(1, 468) = 79.13, *p* < .001, η^2^_p_ = 0.145, sleep quality, *F*(1, 468) = 25.35, *p* < .001, η^2^_p_ = 0.05, and life rhythm, *F*(1, 468) = 31.83, *p* < .001, η^2^_p_ = 0.06. There has therefore been an improvement since the start of the health crisis, with participants feeling happier, sleeping better, and living a more regular lifestyle. When we compared the participants’ responses at *T*3 (during the last lockdown) and *T*4 (one year after), we still found a significant improvement for happiness and sleep (*M*_*T*3- Happiness_ = 4.23, *SD* = 1.383, *M*_*T*4-Happiness_ = 4.59, *SD* = 1.375, *t*(468) = 6.12, *p* < .001, *d* = 0.28; *M*_*T*3-Sleep_ = 4.38, *SD* = 1.61, *M*_*T*4-Sleep_ = 4.55, *SD* = 1.65, *t*(468) = 2.50, *p* = .01, *d* = 0.116). However, life rhythm stabilized as early as *T*3 (*M*_*T*3-Rhythm_ = 4.96, *SD* = 1.509), as no significant difference was observed between *T*3 and *T*4 (*M*_*T*4-Rhythm-_ = 5.06, *SD* = 1.51), *t*(468) = 1.56, *p* = .072.

There was also a significant linear improvement regarding boredom, *F*(1, 468) = 65.94, *p* < .001, η^2^_p_ = 0.12, which was due to a strong decrease in boredom between *T*4 (outside of lockdown) (*M*_*T*4-Boredom_ = 2.64, *SD* = 1.59) and *T*3 (during lockdown) (*M*_*T*3-Boredom_ = 3.34, *SD* = 1.79), *t*(468) = 8.97, *p* < .001, *d* = 0.41. For boredom, no difference was observed between *T*1, *T*2 and *T*3 (all *p*s > .05), although the lockdown rules became less restrictive from one lockdown to the next [see also 16].

Similarly, a linear effect of the survey time factor was observed for anxiety scores (S-STAI), *F*(1, 456) = 20.72, *p* < .001, η^2^_p_ = 0.04, with a significant decrease in anxiety symptoms between *T*4 (*M*_*t*4-Stait_ = 12.15, *SD* = 4.63) and *T*3 (*M*_*t*3-Stait_ = 13.02, *SD* = 4.76) (*t*(456) = 4.66, *p* < .001, *d* = 0.22).

**Fig 5:**
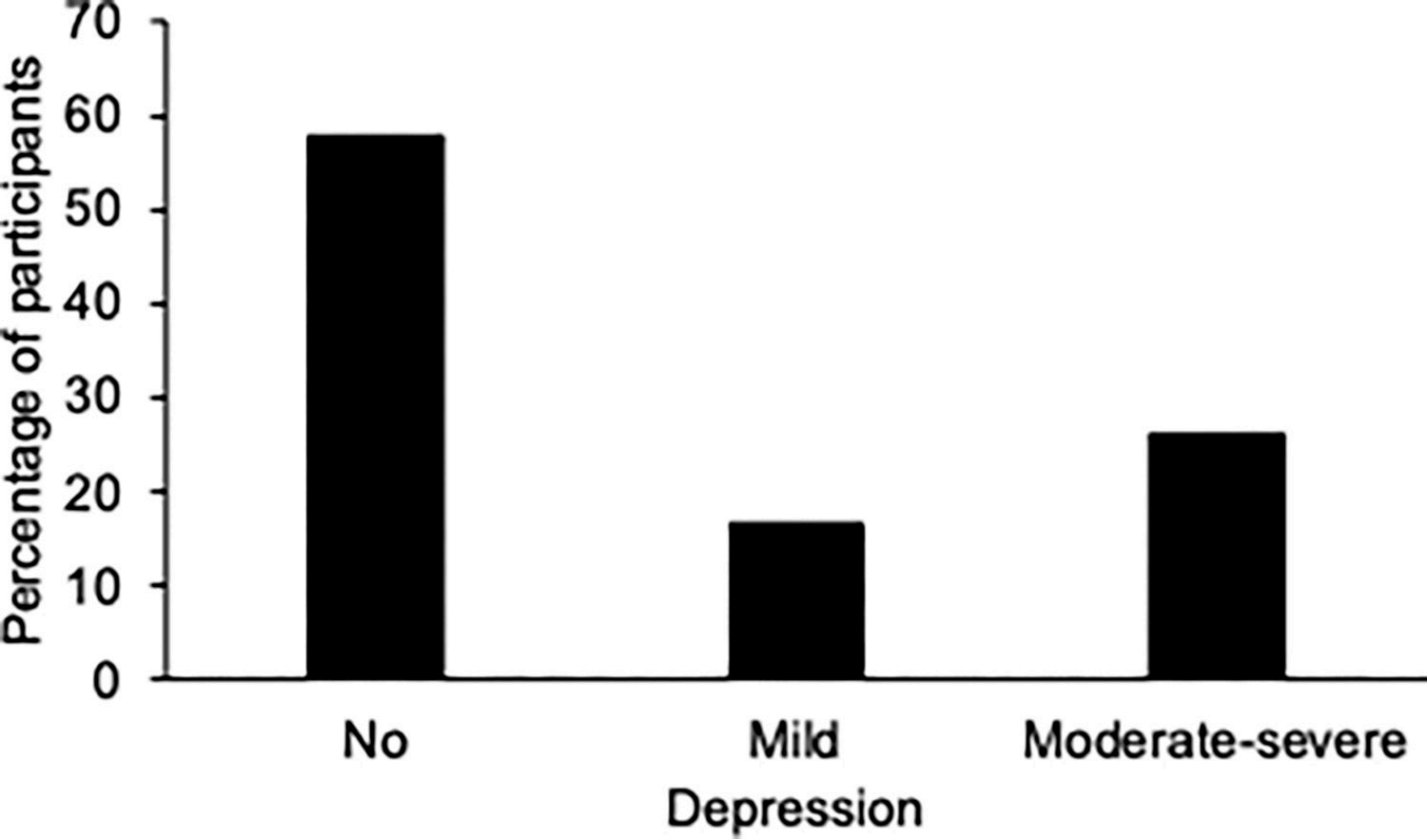
Depression scores. Percentage of participants with no depression, mild and moderate-severe depression 2 years after the beginning of the COVID-19 pandemic (outside of lockdown).

Only depression scores (BDI) did not change significantly across surveys despite a downward trend, *F*(3, 1365) = 2.51, *p* = .06, η^2^_p_ = 0.005 (linear effect, *F*(1, 455) = 0.017, *p* = .897). Indeed, no difference in depression scores was observed between *T*4 (*M*_*T*4-depression_ = 5.16, *SD* = 5.89) and *T*3 (*M*_*T*3-depression_ = 5.46, *SD* = 5.80), *t*(455) = 1.14, *p* = .25, *d* = 0.05, i.e., one year after the last lockdown. At *T*4, the percentage of participants suffering from moderate or severe depression was indeed particularly high, i.e., 25.9% (moderate depression = 19.1%, severe depression = 6.8%), when compared to 16.4% with mild depression and 57.7% with no depression (Fig 5).

**Fig 6:**
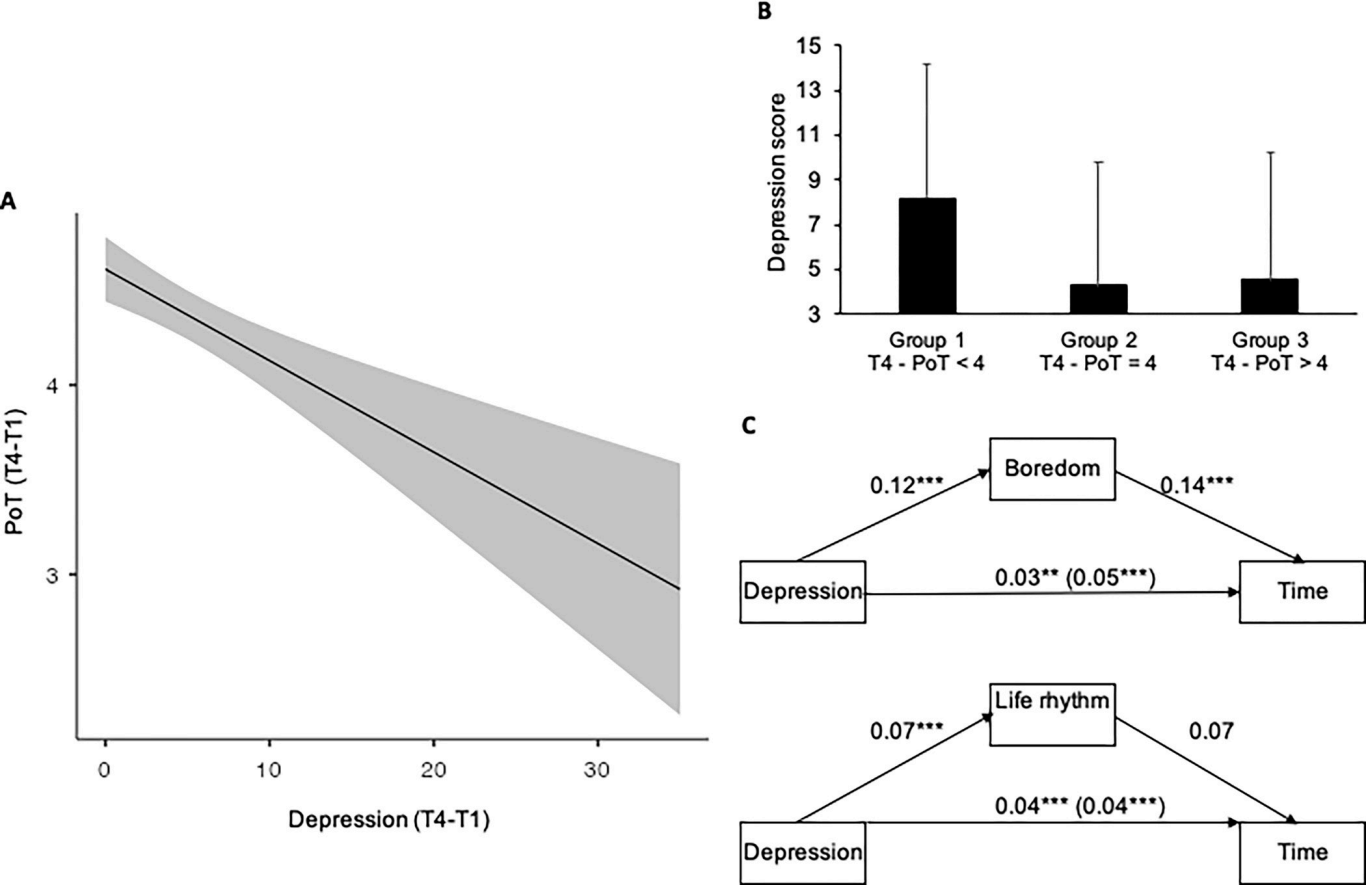
Depression and passage of time. (A) Relationship between the difference scores between the last survey (T4, out of lockdown) and the first survey (T1, first lockdown) for Passage-of-Time judgment and depression; (B) Mean (SD) depression score for participants who, 2 years after the beginning of the Covid-19 pandemic, i.e., at T4 in June 2022, judged that the passage of time was slow (< 4) (group 1), neither fast nor slow (= 4) (group 2) and fast (> 4) (group 3); (C) Mediation analyses.

Because participants were still experiencing a slower passage of time after two years of the health crisis and also because there was no improvement in depression scores, despite significant improvements in other factors (i.e., boredom, happiness, sleep, life rhythm, anxiety), we performed a regression analysis to examine the relationship between persistent depression scores and the persistent feeling of time passing more slowly than usual. This analysis, which was based on difference indexes (T4 score minus T1 score), showed that the more the depression scores increased, the slower time was perceived to pass (*E* = -0.042, *ES* = 0.016, β = -0.12, 95%CI[-0,074; -0,01], *t* = 2.589, *p* = .01 (Fig 6A). Therefore, the feeling of a slowing-down of time persisted two years after the onset of the health crisis and was associated with persistent depression scores, indicating the emergence of a chronic depressive state in a significant number of individuals. In line with this finding, the participants in the group with PoT score lower than 4 (group 1, PoT < 4) were more depressed (*M* = 8.16, *SD* = 5.96) than the participants in the other two groups (group 2, PoT = 4, *M* = 4.25, *SD* = 5.52; group 3, PoT > 4, *M* = 4.52, *SD* = 5.73 (Fig 6B) (*t*(265) = 5.36, *p* < .001, *d* = 0.69; *t*(265) = 4.95, *p* < .001, *d* = 0.63, respectively).

It is clear that the different factors studied in our survey were intertwined, with depressive people being more bored, less happy, sleeping less well, and having a less regular life rhythm. Consequently, the *T*4-*T*1 difference in the scores for the other factors continued to be a significant predictor of the *T*4-*T*1 difference in the PoT judgment (all *p* > .05), as observed in previous studies [16]. Furthermore, when all these factors were included in the same linear regression model, boredom and life rhythm remained the best predictors of individual differences in the present PoT judgments (b = -0.09, *ES* = 0.04, β = -0.11, 95%CI[-0.17; -0,01], *t* = -2.23, *p* = .026; *b* = 0.12, *ES* = 0.05, β = 0.13, 95%CI[0.028; 0.213], *t* = 2.55, *p* = .011). The other factors lost their predictive power (all *ps* > .05) (Table 1). Indeed, the mediation analyses showed that, at *T*4, the total effect of the depression scores on the present PoT judgment was partly (37%) mediated by the indirect effect of boredom (*E* = -0.0179; *SE* = 0.00587, 95%CI[-0.0294, -0.00639], *Z* = -3.05, *p* = .002), although the direct effect of depression on PoT judgment remained significant (E = -0.03; SE = 0.01, 95%CI[-0.05, -0.01], *Z* = -2.46, *p* < .001) (Fig 6C). By contrast, the indirect effect of life rhythm was not significant (E = -0.005; SE = 0.003, 95%CI[-0.01, 0.002], Z = -1.44, *p* = .15) (Fig 6). Therefore, depression and the underlying emotion of boredom were linked to a feeling of a slowing-down of time in the present that persists in a significant number of people two years after the onset of the COVID-19 health crisis.

**Table 1:** The predictors of PoT judgment (*T*4 *score—T*1 *score*). Linear regression model including all significant predictors.

	B	ES	Beta	95%CI		*t*	*p*	R
(Constant)	-0.13	0.091		-0.31	0.049	-1.42	0.155	
Sleep	0.056	0.056	0.054	-0.05	0.166	1.01	0.313	
Life Rhythm	0.121	0.047	0.125	0.028	0.213	2.551	0.011*	
Boredom	-0.09	0.04	-0.11	-0.17	-0.01	-2.23	0.026*	
Happiness	0.122	0.064	0.099	-0.01	0.247	1.91	0.057	
Anxiety	-0.03	0.02	-0.08	-0.07	0.009	-1.54	0.125	
Depression	-0.01	0.017	-0.02	-0.04	0.027	-0.41	0.686	0.293***

### Memory of the passage of time experienced at each lockdown

**Fig 7:**
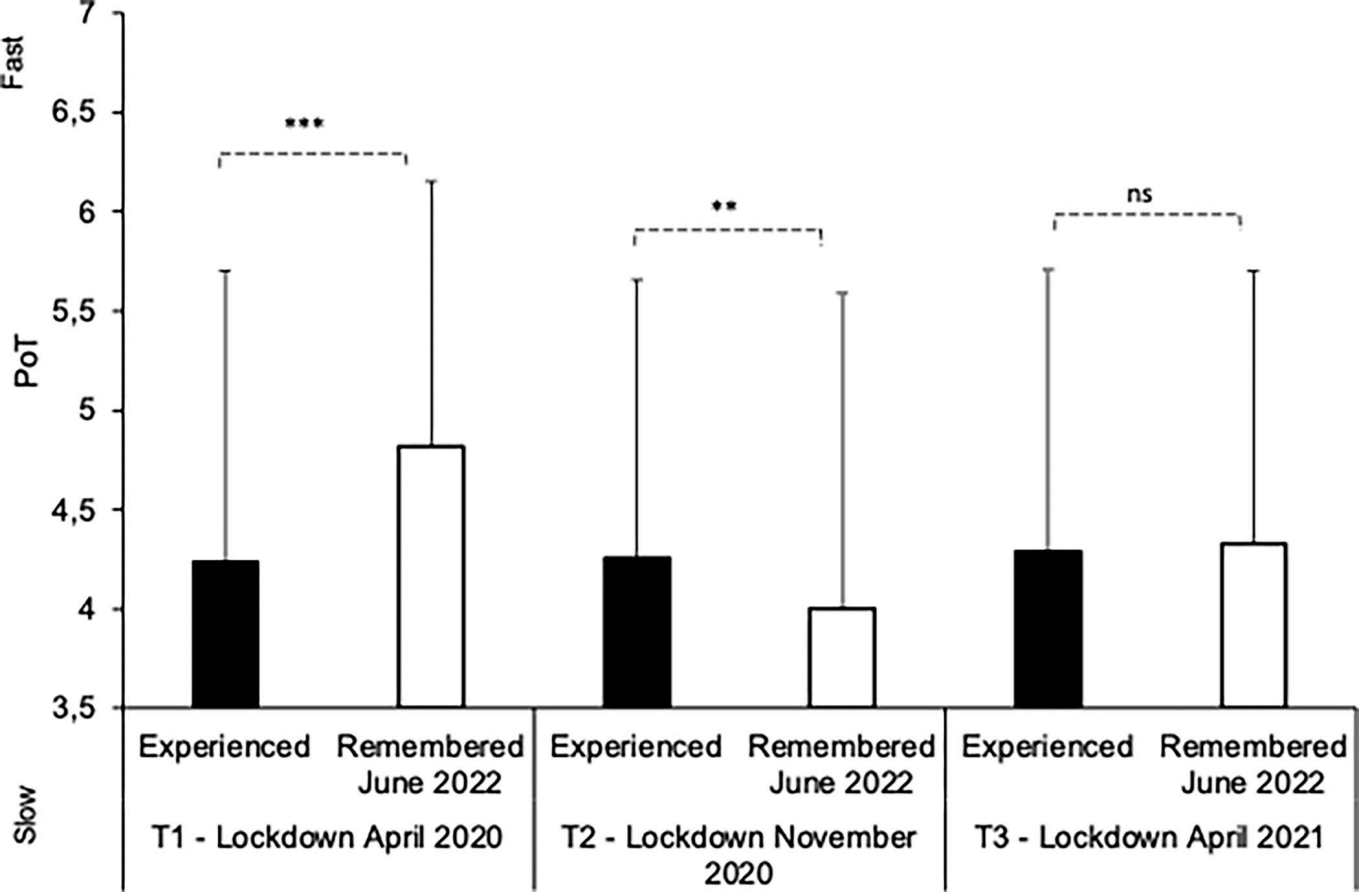
Memory of passage of time. Passage of Time experienced during each of the 3 lockdowns in April 2020 (*T1*), November (*T2*), and April 2021 (*T3*), and Passage of Time for each lockdown remembered at T4 (June 2022), i.e., 2 years after the beginning of the pandemic.

Fig 7 shows the PoT actually experienced at each lockdown (*T*1, *T*2, *T*3) and the recall at *T4* (June 2022) of this experienced PoT. An ANOVA was performed on the PoT judgment with two within-subjects factors: 1) survey time (*T1*/*T2*/*T3*), and 2) experienced/remembered PoT. The ANOVA found a significant interaction between these two factors, *F*(2, 936) = 33.43, *p* < .001, η^2^_p_ = 0.07, implying a significant main effect of survey time, *F*(2, 936) = 34.67, *p* < .001, η^2^_p_ = 0.07, and of experienced/remembered PoT, *F*(1, 468) = 5.06, *p* = .025, η^2^_p_ = 0.01. This significant interaction indicates a distortion of remembered time compared to experienced time at *T*1 (*M*_PoT-remembered_ = 4.82, *SD* = 1.33; *M*_PoT-experienced_ = 4.24, *SD* = 1.469; *t*(468) = 6.68, *p* < .0001, *d* = 0.309), and *T*2 (*M*_PoT-remembered_ = 4, *SD* = 1.59; *M*_PoT-experienced_ = 4.26, *SD* = 1,405; *t*(468) = 3.09, *p* = .002, *d* = 0.143), but not at *T*3 (*M*_PoT-remembered_ = 4.33, *SD* = 1.374; *M*_PoT-experienced_ = 4.29, SD = 1.428; *t*(468) = 0.55, *p* = .055, *d* = 0.03), i.e., the time of the most recent and least restrictive lockdown. The time distortion in memory was therefore greater at *T*1 than at *T*2 (remembered PoT), with the time compression (time judged faster) being greater for the oldest memories, *t*(468) = 7.246, *p* < .001, *d* = 0.335.

Additional regression analyses indicated that participants with higher depression and anxiety scores at *T*4 were more prone to temporal distortion in memory, remembering the PoT during the first lockdown (*T*1 –April 2020) as being faster than it actually was (b = -0.053, ES = 0.16, β = -0.154, 95%CI[-0,084; -0,022], *t* = -3.32, *p* < 0.001; b = -0.082, ES = 0.02, β = -0.186, 95%CI[-0,121; -0,042], *t* = -4.05, *p* < 0.001, respectively).

### Memory of predictive factors experienced during each lockdown

**Fig 8:**
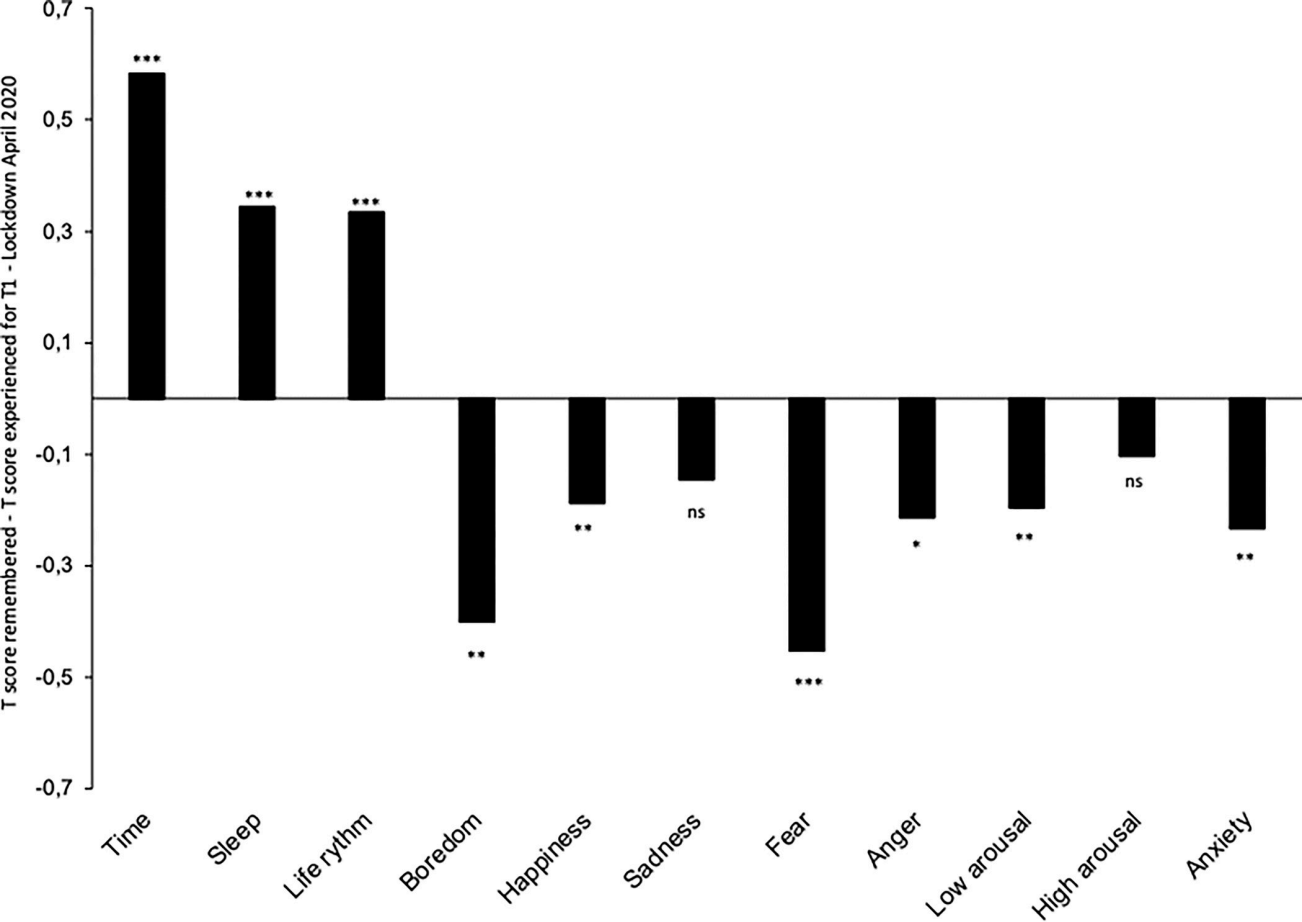
Memory of judgments. Differences between the experience of various factors during the first lockdown as recalled 2 years after the first lockdown and the experience actually reported during the first lockdown.

The difference between current memories (in June 2022, 2 years after the first lockdown) and what was actually experienced during the first lockdown (*T*1—April 2020) was also investigated for the predictive factors of PoT (e.g., sleep, happiness) (Fig 8). A general positivity bias in memory for most of the factors was observed. Indeed, as reported above, participants remembered that time went faster than they actually experienced it at the time. However, they also said that they had slept better (*M*_Sleep-remembered_ = 4.47, *SD* = 1.67; *M*_Sleep-experienced_ = 4.21, *SD* = 1.76; *t*(470) = - 4.796 *p* < .001), that their life rhythm had been more regular (*M*_Rhythm-remembered_ = 4.86, *SD* = 1.59; *M*_Rhythm-experienced_ = 4.53, *SD* = 1.76; *t*(470) = - 4.09, *p* < .001), and that they had felt less boredom (*M*_Boredom-remembered_ = 3.05, *SD* = 1.81; M_Boredom-experienced_ = 3.46, *SD* = 1.88; *t*(470) = 4.42 *p* < .001. By contrast, they reported being less happy than they actually were (*M*_Happiness-remembered_ = 3.82, *SD* = 1.59; *M*_Happiness-experienced_ = 4.10, *SD* = 1.48, *t*(472) = 2.92, *p* = .004).

In Fig 8, we added the other emotions initially tested in Martinelli et al.’s study [3], and found the same positivity bias for fear, *M*_Fear-remembered_ = 3.59, *SD* = 1.799; *M*_Fear-experienced_ = 3.90, *SD* = 1.77; *t*(472) = 5.12 *p* < .001), anger (*M*_Anger-remembered_ = 3.40, *SD* = 1.80; *M*_Anger-experienced_ = 3.54, *SD* = 1.83; *t*(472) = 2.39 *p* = .017), and anxiety *M*_Anxiety-remembered_ = 3.81, *SD* = 1.79; *M*_Anxiety-experienced_ = 3.91, *SD* = 1.75; *t*(472) = 2.77 *p* = .006). The only measures for which there was no difference were sadness and high-arousal level (*p*s > .05), and participants remembered being less calm than they actually were (*M*_Low-Arousal-remembered_ = 3.59, S*D* = 1.57; *M*_Low-Arousal-experienced_ = 3.80, *SD* = 1.54, *t*(472) = 2.73, *p* = .007).

This positive memory bias is observed regardless of individual depression and anxiety scores for most factors, as suggested by the regression analyses on the memory distortion index at *T*1 with the depression scores as variable. However, the more depressed participants were, the more likely they were to report sleeping better during the first lockdown than they actually did (*b* = 0.028, *ES* = 0.012, β = 0.106, *t* = 2.26, *p* = .02). Similarly, the more anxious they were, the more likely they were to report that they were less sad, less fearful, less angry, and less anxious than they actually were (*b* = -0.054, *ES* = 0.018, β = -0.144, *t* = -3.102, *p* = .002; *b* = -0.039, *ES* = 0.019, β = -0.096, *t* = -2.07, *p* = .039; *b* = -0.069, *ES* = 0.019, β = -0.172, *t* = 3.33, *p* < .001; *b* = -0.039, *ES* = 0.017, β = -0.104, *t* = -2.22, *p* = .27).

Furthermore, the compression of PoT memories was significantly correlated with the positive bias in the memory representation of different affects experienced during the first lockdown (happy, *R* = -0.233; boredom, *R* = -0.30; sleep, *R* = -0.46; life rhythm, *R* = -0.12, all *p* < .01).

## Discussion

Our study enabled us to survey the same participants several times over the course of two years and examine changes in their judgment of the passage of time since the beginning of the COVID-19 pandemic. Moreover, their answers about memories of PoT experienced before the pandemic and during each lockdown period–which were directly compared to the PoT judgments actually made during these lockdown periods–raise new questions about the processes involved in the recall of the passage of time for specific past events and their role.

### Many people still felt a slowdown in the passage of time 2 years after the beginning of the COVID-19 pandemic

Our results showed that 2 years after the beginning of the pandemic, and in the absence of any lockdown or health measures, the passage of time was not judged to have returned to its initial and usual pace in the French population. Indeed, the average score revealed a persistent feeling of time slowing compared to before the pandemic, with the result that there was no significant difference between the PoT judged during the different prior lockdowns and out of lockdown, i.e., 2 years after the first lockdown. In particular, in 2022, 42.1% of participants considered that time was passing fast, while the corresponding level in 2020, before the pandemic, was 69.2%. Similarly, in 2022, 20.3% said that the passage of time had slowed down considerably (PoT < 4 on a 7-point scale), whereas such a view was expressed by only 6.7% of them before the pandemic. These results obtained in the French population are consistent with those of studies conducted in other countries (Iraq, Germany, UK) showing that a significant percentage of people report a temporal lengthening for a long period going from 8 to 14 months as of the beginning of the pandemic [5, 7, 17, 18]. However, these studies reported the retrospective judgment of PoT over a long period (including the lockdowns), and not the judgment of PoT in the present as was assessed in our study. Consequently, our research extends and complements these studies by showing that, even now when life has returned to normal and no further health-related restrictions are in force, a large number of people are still experiencing a significant slowing-down of time.

### The persistence of the feeling of a slowed-down time associated with individual internal factors, i.e., depressive state

We suggested that the persistence of a slowed-down time two years after the beginning of the COVID-19 pandemic and in the absence of any lockdown might be linked primarily to internal contextual factors, independently of external factors specific to the current activity or living conditions [13, 14]. Our results are consistent with this hypothesis in demonstrating that the persistence of slow time outside of lockdown was associated with a general depressive state, and not with factors (boredom, less happiness, disruption of sleep quality, loss of life rhythm) initially identified to be significant predictors of PoT judgment in the surveys conducted during the previous lockdowns [2, 3]. This confirms that the factors on which PoT judgments are based are varied and context-sensitive, i.e., internal context specific to the individual, or external contexts linked to the unfolding of events in a specific environment [13, 15]. The previous surveys were all conducted during a lockdown period, with the result that the predictors of PoT during this period directly reflected the specific life conditions experienced by the participants at the time of the surveys (e.g., boredom, disruption of sleep quality). When the lockdowns ended and life resumed its normal course, with the usual freedoms and a wider range of physical and social activities, an improvement was observed for these factors, as evidenced by the results of our study outside of lockdown. Therefore, the feeling of a slowed-down time, which persists despite the improvement of these factors, can no longer be linked to them because they have improved. As our results show, this perception is then primarily due to a mood disorder which is characteristic of depressive symptoms that exist regardless of the improvement in living conditions.

In their study conducted in the United Kingdom, Ogden and Piovesan [18] also observed that the more depressed people were, the more they considered that the 12 months that passed since the first lockdown were particularly long. Like us, they therefore conclude that depression has become the major predictor of a slowing-down of time after the lockdown periods. Obviously, our results indicated that other factors, namely boredom and loss of usual life rhythm, remained significant predictors of PoT judgment. However, these factors played a lesser role and are associated with a depressed emotional state. Before the studies relating specifically to COVID-19, it was already known that depressed people experience a slowing of time in their daily lives. In addition, several studies have shown that quarantine has a long-lasting effect on mental health [e.g., 20, 30, 31] and that the number of people suffering from depression has increased since the COVID-19 health crisis and the restrictive protective measures [32, 33]. Our study on the PoT judgment two years after the beginning of the pandemic is thus entirely consistent with these studies.

The more detailed analysis of our data reveals that the participants who still experienced a considerable slowing of time 2 years after the beginning of the COVID-19 pandemic (T4) had temporal judgments similar to those of the others before the first lockdown. However, as of the first lockdown (T1), they described a slower flow of time than the others did. This suggests some inter-individual variability in the psychological effects of the lockdown, with some people experiencing confinement less well than others. It has been shown that fragile people with pre-existing mental health problems were particularly vulnerable to the effect of the quarantine [20]. Depressed people expressed more negative affects (boredom, sadness) during the first COVID-19 lockdown than others [34]. During the first lockdown, they also suffered more from social isolation and disruption of their life rhythm and the associated effects on sleep [34]. Moreover, studies have shown that people who, for various clinical reasons, are psychologically vulnerable experienced a greater expansion of time during the first lockdown than control participants [35, 36]. As suggested by Lau et al. [35], pre-existing health issues have intensified the negative effects of the lockdowns in a way which resembles “a magnifying glass effect” (page 9). Therefore, the feeling of a slowing-down of time as much as 1 to 2 years after the periods of lockdown could be specific to a psychologically fragile category of participants. And the number of people suffering from depressive symptoms is particularly high in socio-economically highly developed European countries such as France and Germany [37].

One limitation of our study is that we did not perform (like most survey studies) any clinical assessment of the participants before the pandemic. However, psychological issues may also only have arisen when people were confronted with a very difficult situation, such as the lockdown, that they had never encountered before and which they experienced as traumatic. In any case, like other studies on time, our study, which was conducted during the COVID-19 pandemic, clearly reveals that the PoT judgment is highly sensitive to changes in mood.

### Memory time compression for past lockdown periods

In our study, we assessed not only the judgment of the PoT in current life but also the memory representation of past PoT for the 3 real-life periods of lockdown which had occurred one or two years previously. The originality of our data is that they clearly indicate that the PoT accelerates in episodic memory for the oldest memories, with the result that the past PoT was recalled as having been faster than it actually was. As predicted, this acceleration of PoT resembles the phenomenon through which events become temporally compressed in episodic memory [25–27]. This accounts for the fact that the recall of the unfolding of an event experienced in the past is shorter than the actual timecourse of the event experienced in reality. This phenomenon is still poorly understood and several alternative explanations have been proposed. Nevertheless, various sources of data converge to explain it in terms of the recall of an unfolding event as a succession of moments, some of which are omitted or forgotten. Thus, the fact that the PoT during the first lockdown was recalled as being faster than it actually was could be due to the forgetting of certain elements of previously experienced events (e.g., forgetting of episodes of boredom or of their frequency). This would be consistent with the theory of retrospective judgment of durations [38, 39], according to which the length of the remembered duration of a past event depends on the amount of non-temporal information recalled, with duration being retrospectively judged shorter when less information is recalled.

Somewhat surprisingly, and as noted in the introduction, longitudinal COVID-19 studies of PoT have found not a shortening but a lengthening of time [5, 7, 18]. This may be due to the type of PoT judgment used, i.e., retrospective judgments of the PoT over a long period of several months instead of the memory of the present-PoT judgment examined in our study. It may also be due to the recency of the period to be judged. In our study, the acceleration of the PoT was significant for the oldest memories (first lockdown) and not for the most recent ones (*T*2, *T*3). The compression of time depends on the characteristic of events experienced in the past (goal-oriented, emotion) [40, 41]. We therefore consider that temporal distortion occurs in long-term memory for long-past emotional events, such as the experience of lockdown, rather than for neutral events. It is indeed well known that emotional events are remembered better [42]. In a similar vein, Cocenas et al. [43, 44] showed that memories for the duration of threatening stimuli are more accurate than for that of neutral stimuli.

Another hypothesis, which does not exclude the one proposed above, is that the faster PoT for past events retrieved from long-term memory results from a process of reconstruction in autobiographical memory (when memories are not too recent and vivid). Autobiographical memory is constructed from both episodic events and from semantic knowledge about the events and the self (self-schema) [45]. As a result of coping strategies or to present a positive self-image, individuals often tend to judge past events and their behaviors more favorably than they actually were. This positivity bias was observed for most of the factors examined in our studies. Indeed, participants reported sleeping better during the first lockdown than they did in reality. They also reported having a more regular life rhythm and feeling less bored, for example. In addition, the distortion of the memory representation of PoT (faster time) compared to the actual PoT was correlated with the positivity bias for the other factors. Therefore, retrospective judgment of past PoT would also seem to be influenced by the mood and/or decision at the time of recall. This highlights the complexity of the processes involved in the PoT judgment.

In our study, the positivity bias was observed in the majority of participants regardless of their level of depression or anxiety. However, the temporal distortion in memory tended to be greater in depressed and anxious people. In addition, the more depressed people were, the better they reported sleeping, and the more anxious they were, the more likely they were to report being less sad, less fearful, less angry and less anxious than they actually were during the first lockdown. Thus, the positivity bias that affects memory for time would tend to be greater in people with depressive and anxious symptoms. In sum, post-pandemic depression and anxiety tended to exacerbate distortions in the memory representations of time, of affects and of contextual factors.

In conclusion, our study shows that the COVID-19 pandemic has a long-lasting effect on the judgment of PoT by tending to induce a sensation of a slowing-down of time, which is itself related to the greater number of people suffering from depressive symptoms in the general population in France following the lockdown measures. However, 2 years after the beginning of the pandemic crisis, psychological processes of reconstruction in memory occurred, with the result that the past PoT experienced during the first lockdown was judged as having been faster than it actually was, especially among depressed and anxious people. This could be related to a positivity bias or forgetting in memory. Further studies are now needed to better investigate this phenomenon of PoT acceleration in long-term memory and the underlying mechanisms, a yet under-investigated field of research.

Our study confirms that PoT judgment is highly sensitive to changes in external and internal contexts (e.g., traumatic events, boredom, sadness, depression). It would therefore be useful in a clinical approach to construct a test based on time judgment, which is easy to collect regularly, and which would be a good way to detect an emerging psychological problem, such as depression. Our study also shows the long-term consequences of the government’s management of the Covid-19 pandemic, namely the installation of a chronic depressive state (expressed by a slowing down of time) in a significant part of the population, which nevertheless remains mild to moderate. However, this population must be treated now to avoid more severe depression. It is also not advisable to lock the population up again in the event of a new health crisis. Their psychological fragility, which has increased with the previous lockdowns, would no longer allow them to cope and could cause them to fall into a severe depression, which is difficult to treat.

## Supporting information

S1 Data Mean data for Figs 1–8. (XLSX)

S1 Dataset The minimal anonymized data set. (XLSX)
